# Phantom-derived method for improving accurate material decomposition in photon-counting detector CT

**DOI:** 10.1186/s41747-026-00705-2

**Published:** 2026-04-02

**Authors:** Sam Springer, Bibi Martens, Thomas Flohr, Cécile Jeukens

**Affiliations:** 1 Department of Radiology and Nuclear Medicine, Maastricht University Medical Centre, Maastricht, The Netherlands; 2 Siemens Healthineers, Forcheim, Germany

**Keywords:** Iodine quantification, Iron quantification, Material decomposition, Phantom study, Photon-counting detector CT

## Abstract

**Objective:**

Photon-counting detector CT (PCD-CT) enables spectral imaging with material separation. Accurate iodine and iron quantification remains challenging due to inevitable low- and high-energy base material CT number mismatches and dual-energy ratio (DER) variability. This study develops and validates a correction method addressing these issues to improve iodine and iron quantification in PCD-CT.

**Materials and methods:**

A spectral CT abdomen phantom containing rods with known iodine (0.5–15.0 mg/mL) and iron (2.0–25.0 mg/mL) concentrations in water- and liver-equivalent material was scanned on a clinical PCD-CT under varying tube voltages, dose levels, and with/without a fat ring. High- and low-energy CT numbers of base materials and DER values were inputs for the correction method. Material concentrations calculated with and without correction were validated against known phantom values.

**Results:**

The correction method significantly reduced quantification errors. Iodine errors fell below 5% for concentrations ≥ 2 mg/mL and iron errors below 15% for concentrations ≥ 5 mg/mL. Without correction, errors reached up to 83% (iodine) and 85% (iron) at low concentrations, reduced to 23% and 47%, respectively, after correction.

**Conclusion:**

The proposed correction method improves accuracy in spectral material decomposition for PCD-CT, supporting its potential for better clinical assessment of lesion contrast enhancement, therapy response and hepatic burden evaluation.

**Relevance statement:**

This technical note introduces a phantom-based correction method for photon-counting detector CT that improves iodine and iron quantification by addressing base material Hounsfield Unit (HU) mismatches and dual-energy ratio variability. The method reduces quantification errors and offers a practical calibration procedure, supporting the potential for clinically reliable iodine and iron quantification.

**Key Points:**

- PCD-CT correction method reduces concentration errors across varying scan protocols and configurations.
- Implementation guide supports adaptation to other scanners.
- Accurate iodine and iron quantification supports diagnosis and treatment assessment.

**Graphical Abstract:**

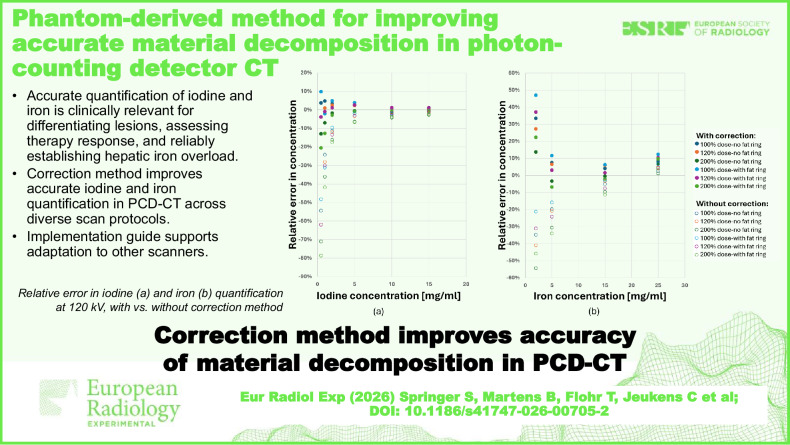

## Introduction

In Western countries, hemochromatosis, an iron overload disorder of the liver, is often diagnosed using MRI and defined as a liver iron content greater than 36 μmol/g. Hepatic iron overload can progress to end-stage liver disease, which is associated with an increased risk of developing hepatocellular carcinoma [1]. Screening for hepatic iron content may therefore be useful in early diagnostics. Additionally, iodine is commonly used as a contrast agent in CT imaging. Accurate iodine quantification can aid in differentiating tumor types and assessing therapy response [2–6].

Photon-counting detector CT (PCD-CT) has revolutionized quantitative CT imaging by offering improved spatial resolution, reduced electronic noise, improved image contrast and spectral information capture [7, 8]. This could be clinically relevant, as PCD-CT holds great promise for more precise iodine and iron quantification [9–11].

However, accurate material decomposition depends on system-specific spectral properties. Across all PCD-CT systems, differences between expected and measured base material CT numbers in the various energy bins can propagate into quantification errors. These effects arise from unavoidable tolerances in scanner calibration and are present in every CT system; for example, AAPM guidance for diagnostic CT scanners accepts a tolerance of ±5 HU for water, reflecting internationally accepted quality control standards [12].

This technical note proposes a user-side vendor-independent correction method to improve iodine and iron quantification using PCD-CT, without replacing vendor calibration. A classic two-material decomposition based on data from two energy bins was used as an example. By correcting for the difference in low- and high-energy base material CT numbers arising from residual calibration errors and dual-energy ratio (DER) variability, a more robust quantification method is established. A step-by-step implementation guide is provided.

## Materials and methods

### Phantom

A spectral CT abdomen phantom (QRM) was used with cylindrical holes (20 mm) filled with rods containing iodine in water-equivalent or iron in liver-equivalent material. Iodine concentrations were 0.5, 1.0, 2.0, 5.0, 10.0, and 15.0 mg/mL; iron concentrations were 2.0, 5.0, 15.0, and 25.0 mg/mL. Both configurations were scanned with and without a fat ring, resulting in phantom sizes of 250 × 350 mm and 200 × 300 mm, respectively.

### CT acquisition parameters

Images were acquired using a standard abdominal CT protocol on a clinical PCD-CT (NAEOTOM Alpha, Siemens Healthineers AG). Multiple scans were made with different scan parameters:

- 120 kV and 140 kV
- 100%, 120%, and 200% doses. The CTDI_vol_ at 100% dose was 4.5 mGy.
- Collimation width = 144 × 0.4 mm
- Revolution time = 0.5 s
- Pitch = 0.8

Images were reconstructed with 2 mm slice thickness using the Qr40 kernel and quantum iterative reconstruction (QIR) level 3 and saved as spectral post-processing (SPP) images for analysis in Syngo.via (Siemens Healthineers).

### Image analysis

#### *Base material CT numbers*

In Syngo.via, the images were analyzed using the ‘Virtual Unenhanced’ application, which provides material (iodine) images and virtual non-contrast images based on a two-material decomposition into water and iodine. By changing the pre-set DER for iodine, other material images, such as iron, can be generated. Regions of interest (ROIs) of 4 cm^2^ were drawn inside the rods, and the attenuation (HU) of the rods in the low- and high-energy images was measured. For all images, the attenuation in the low and high-energy images (in HU) was plotted against iodine or iron concentration. Low- and high-energy base material CT numbers ($L{E}_{{base}}$ and $H{E}_{{base}}$) were defined as the y-intercepts of linear fits of CT number versus concentration plots, and ${\Delta }_{{\rm{base}}}$, their difference, was calculated.

#### *Dual-energy ratio*

To determine the DER of each phantom configuration and CT acquisition parameter set, the high-energy HU were plotted against the low-energy HU and a linear fit was made through these data points. The slope of this fit equalled the ${\mathrm{DER}}$ for that specific configuration.

#### *Conversion from contrast media to concentration*

**Fig. 1:**
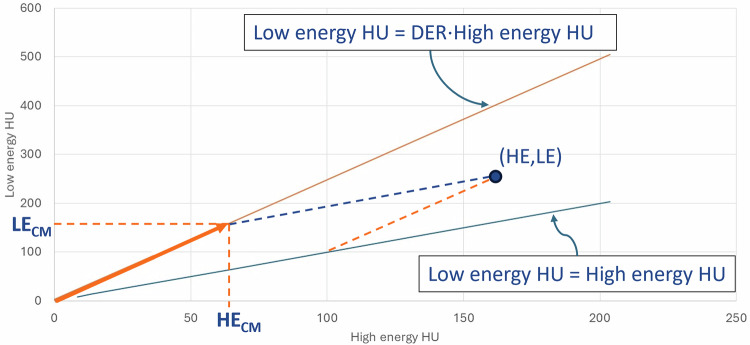
Visualization of the determination of the low-energy contrast material (LE_CM_) and high-energy contrast material (HE_CM_) voxel values (unit = Hounsfield Units (HU)), which are used to calculate the concentration map in Syngo.via. Two solid lines are shown: one blue line with a slope equal to 1 (the water line representing all image voxels containing only water-equivalent tissues of different density: low energy HU  = high energy HU), and one orange line with a slope equal to the DER (the material line representing all image voxels containing only the chosen material at different concentrations: low energy HU = DER $\cdot$ high energy HU). The point (HE, LE) represents a measured image voxel containing a mixture of water and the material. This point is projected onto the material line along a line parallel to the water line (the blue dotted line). The intersection point (HE_CM_, LE_CM_) corresponds to the material-specific component of the voxel with measured high- and low-energy CT-numbers (HE, LE)

The Virtual Unenhanced application in Syngo.via calculates a material image, *i.e.*, a concentration map (CM), based on a selected DER specific to the material (*e.g.*, iodine, iron). The calculation is based on a standard two-material decomposition in image space [13] and thus projects the measured voxel CT numbers (*HE*, *LE*) onto a material line with a slope equal to the DER. A visual representation of this projection geometry is shown in Fig. 1. The value of a voxel in the calculated CM generated by Syngo.via represents the attenuation in HU of the material at the given concentration in a 70 keV virtual monoenergetic image (VMI). To improve the accuracy of the CM, a correction is applied to account for differences in the low- and high-energy CT numbers of the base material (${\Delta }_{{\rm{base}}}$) and for variability in the DER. Additionally, a concentration scaling factor ($\alpha$) translates HU values in the CM to real concentrations (mg/mL). The core equations for the described concentration calculations are described in Box 1.

Box 1 Core equations for concentration calculation In Fig. 1, the low- and high-energy contributions to the projection on the material line are given by $L{E}_{{CM}}={DER}\cdot \frac{{LE}-{HE}}{{DER}-1}$ and $H{E}_{{CM}}=\frac{{LE}-{HE}}{{DER}-1}$, respectively.

The value of a voxel in the CM is a weighted sum of $H{E}_{{CM}}$ and $L{E}_{{CM}}$:

1 $$C{M}_{{measured}}=w\cdot L{E}_{{CM}}+\left(1-w\right)\cdot H{E}_{{CM}}=\left({DER}\cdot w+1-w\right)\cdot \frac{{LE}-{HE}}{{DER}-1}$$

Where $w$ is a vendor-specific weighting factor that depends on the tube voltage used. The values of *LE* and *HE*, however, do not account for the inherent difference between the low- and high-energy CT numbers of the base material.

To compensate for the difference in base material CT numbers, the equation can be rewritten as:

2 $$C{M}_{{corrected}}=\left({DER}\cdot w+1-w\right)\cdot \frac{L{E}_{{measured}}-H{E}_{{measured}}+{\Delta }_{{\rm{base}}}}{{DER}-1}$$

Where $L{E}_{{measured}}$ and $H{E}_{{measured}}$ are the low- and high-energy HU measured in Syngo.via, respectively.

The values in the CM (in HU) can be translated to real concentrations (in mg/mL) with:

3 $$C=\frac{1}{\alpha }\cdot {CM}$$

For each configuration, the concentration scaling factor $\alpha$ was determined as the slope of a linear fit of known concentrations $C$ versus $C{M}_{{corrected}}$ values calculated using Eq. 2.

#### *Mean method*

For each tube voltage and insert type, a mean method was made by averaging the values of ${\mathrm{DER}}$, ${\Delta }_{{base}}$ and $\alpha$ across all scan configurations per kV and inserting these average values into the previously described concentration calculations. The average values are denoted as *DER*_*avg*_, ${\Delta }_{{base},{avg}}$ and ${\alpha }_{{avg}}$. The accuracy of the mean method was evaluated by comparing the calculated concentrations of the rods in the phantom to the true concentrations. The error in the concentration calculation when using the uncorrected CM values was also calculated. The uncorrected CM values were calculated using *DER*_avg_ and ${\alpha }_{{avg}}$, but not accounting for ${\Delta }_{{base},{avg}}$. The equations describing the concentration correction using the mean method are described in Box 2.

Box 2 Mean method correction for improved concentration accuracy Equation 2 can be rewritten as

4 $$\begin{array}{c}C{M}_{{corrected}}=\left({DER}\cdot w+1-w\right)\cdot \frac{L{E}_{{measured}}-H{E}_{{measured}}}{{DER}-1}+\left({DER}\cdot w+1-w\right)\cdot \frac{{\Delta }_{{base}}}{{DER}-1}\\ =C{M}_{{measured}}+\left({DER}\cdot w+1-w\right)\cdot \frac{{\Delta }_{{base}}}{{DER}-1}\end{array}$$

Where $C{M}_{{measured}}$ is the CM value in the CM map provided by a standard material decomposition (as in the Virtual Unenhanced Application of the Syngo.via) with a specific ${DER}$.

For the mean method, Eq. 4 can be rewritten as:

5 $$C{M}_{{corrected}}=C{M}_{{measured}}+\left({DE}{R}_{{avg}}\cdot w+1-w\right)\cdot \frac{{\Delta }_{{base},{avg}}}{{DE}{R}_{{avg}}-1}$$

Where $C{M}_{{measured}}$ is the CM value in the CM map based on a DER equal to *DER*_*avg*_.

Based on Eq. 5, the concentration using the mean method is given by:

6 $$C=\frac{1}{{\alpha }_{{avg}}}\cdot \left(C{M}_{{measured}}+\left({DE}{R}_{{avg}}\cdot w+1-w\right)\cdot \frac{{\Delta }_{{base},{avg}}}{{DE}{R}_{{avg}}-1}\right)$$

The error in the concentration calculations was given by:

7 $${Error}=\frac{{C}_{{measured}}-{C}_{{true}}}{{C}_{{true}}}\cdot 100 \%$$

Where ${C}_{{measured}}$ is the concentration determined using Eq. 6 and ${C}_{{true}}$ is the true concentration present in the rods.

## Results

### Phantom measurements

In Table 1, the values of ${\Delta }_{{base}}$, DER and $\alpha$ are given for all scan configurations for the iodine in water and the iron in liver inserts, respectively. It can be seen that ${\Delta }_{{base}}$, ${DER}$ and $\alpha$ vary across the configurations.$\,{\Delta }_{{base},{avg}}$, ${DE}{R}_{{avg}}$, ${\alpha }_{{avg}}$ are shown in Table 2. ${\Delta }_{{base},{avg}}$ was larger for iodine in water than for iron in liver, indicating greater deviations between low- and high-energy HU for the water-based inserts. ${\mathrm{DER}}$ and $\alpha$ were stable with small standard deviations, whereas ${\Delta }_{{base}}$ showed slightly higher variability, especially for iodine at 120 kV. These results highlight that tube voltage and material type influence key parameters in the correction model.

**Table 1:** ${\varDelta }_{{base}}$, dual-energy ratio (${\mathrm{DER}}$) and concentration scaling factor $\alpha$ for the iodine in water and iron in liver inserts under different scan configurations in which the kV and dose are varied, and with/without a fat ring

		kV	Dose	${\Delta }_{{base}}$	${DER}$	$\alpha$
Iodine in water	No fat ring	140	100%	-5.72	2.48	26.45
			120%	-5.77	2.48	26.45
			200%	-7.25	2.48	26.43
		120	100%	-5.01	2.08	26.23
			120%	-5.78	2.08	26.17
			200%	-7.45	2.09	26.18
	With fat ring	140	100%	-4.20	2.52	26.94
			120%	-4.95	2.50	26.65
			200%	-6.55	2.50	26.59
		120	100%	-4.47	2.11	26.40
			120%	-5.47	2.11	26.42
			200%	-7.72	2.12	26.33
Iron in liver	No fat ring	140	100%	-3.85	2.18	4.30
			120%	-4.16	2.15	4.30
			200%	-5.77	2.17	4.30
		120	100%	-4.24	1.95	4.03
			120%	-4.58	1.91	4.05
			200%	-5.64	1.92	4.02
	With fat ring	140	100%	-3.39	2.20	4.36
			120%	-3.19	2.22	4.35
			200%	-4.23	2.16	4.32
		120	100%	-2.66	1.93	4.12
			120%	-3.50	1.96	4.07
			200%	-4.64	1.91	4.09

**Table 2:** ${\varDelta }_{{base},{avg}}$, DER_avg_, and ${\alpha }_{{avg}}$ for the iodine in water and the iron in liver inserts, for both 120 kV and 140 kV. Values are presented as mean ± standard deviation

	kV	${\Delta }_{{base},{avg}}$	${DE}{R}_{{avg}}$	${\alpha }_{{avg}}$
Iodine in water	140	-6.16 ± 0.88	2.49 ± 0.02	26.52 ± 0.18
	120	-5.56 ± 1.35	2.10 ± 0.02	26.29 ± 0.11
Iron in liver	140	-4.71 ± 0.81	2.18 ± 0.02	4.32 ± 0.03
	120	-3.60 ± 0.72	1.93 ± 0.02	4.06 ± 0.04

### Error in concentration calculations

**Fig. 2:**
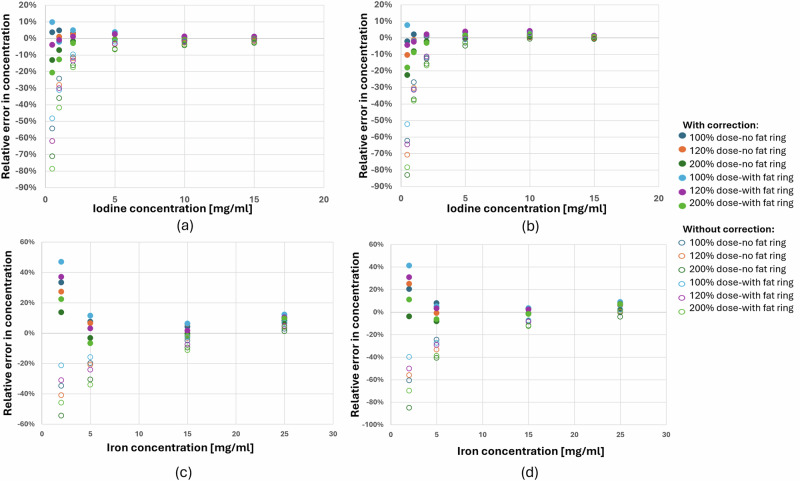
Relative error of the calculated iodine and iron concentrations for different scan conditions, with and without the correction method. **a**, **b **Show the relative error for iodine at 120 kV and 140 kV, respectively. **c**, **d** Show the relative error for iron at 120 kV and 140 kV, respectively

Figure 2 presents the relative errors in iodine and iron concentrations for the different CT configurations, comparing the mean method (Eq. 6) with the uncorrected approach (Eq. 1).

For both materials, the relative error is highest at low concentrations and decreases as concentration increases, regardless of whether corrected or uncorrected CM values are used. For iodine, corrected CM values reduce the maximum error to 23% below 2 mg/mL, versus 83% uncorrected. Above 2 mg/mL, the error remains below 5% using the corrected values, whereas the uncorrected values only achieve this at 10 mg/mL and above.

For iron, corrected CM values result in lower or similar errors up to 5 mg/mL, with a maximum error of 47% using the corrected values versus 85% using the uncorrected values. Above 5 mg/mL, the error remains below 15% using the corrected values, whereas the uncorrected values only achieve this at 15 mg/mL and above.

## Discussion

This study proposed a correction method to address mismatches in low- and high-energy base material CT numbers and DER variability in PCD-CT, aiming to improve iodine and iron concentration measurements. Correcting both sources of error substantially improved quantification accuracy.

Clinically observed iodine concentrations can be low; for example, 0.7–2.7 mg/mL in pancreatic cancers during therapy-response assessment [14]. The proposed method significantly reduces the absolute relative error in iodine quantification in this concentration range.

The clinically relevant transition between normal hepatic iron content and mild iron overload occurs around 1.8 mg/mL [15]. As the proposed method greatly reduced the error for iron quantification at 2.0 mg/mL, the proposed method also shows its potential at clinically relevant iron concentrations.

This enhanced accuracy could have meaningful clinical implications, particularly where differences in iodine or iron concentrations affect diagnostic confidence. By addressing systematic biases in two-material decomposition, our approach represents a step toward improving current PCD-CT quantification workflows.

### Limitations

This study has several limitations. First, the evaluation was conducted on a single PCD-CT scanner using a fixed set of acquisition protocols. Second, only two phantom sizes were included to approximate underweight and normal-weight patients, but additional studies including overweight and obese individuals are needed. Third, because the DER and difference between low- and high-energy base material CT numbers are scanner- and protocol-dependent, centers applying this method should perform the measurements themselves, following the implementation protocol provided in Box 3, to obtain scanner-specific ${\Delta }_{{base},{avg}}$, ${DE}{R}_{{avg}}$ and ${\alpha }_{{avg}}$. Finally, the work represents a proof-of-principle demonstration rather than a full cross-platform validation; future PCD-CT systems were not yet available for testing. While the underlying approach, quantifying system-specific low- and high-energy HU mismatches and correcting for them, is general and transferable to any spectral material-decomposition pipeline, its transferability to other PCD-CT systems remains to be validated once they become available.

### Future perspectives

Accurate quantification of iodine and iron can be clinically relevant for differentiating (liver) lesions, assessing therapy response, and establishing hepatic iron overload at an early stage. Relevant clinical populations include patients at risk of iron overload, such as those with chronic liver disease, and patients in whom iodine quantification supports treatment-response assessment [1–6]. Validation in patient cohorts, with comparison of PCD-CT measurements to MRI and/or biopsy, is therefore essential.

If confirmed *in vivo*, the improved accuracy at lower iodine and iron concentrations could potentially support opportunistic screening. Although PCD-CT is not currently used for screening, routine examinations might, in principle, provide information relevant to early iron overload or subtle iodine uptake differences. Whether such applications are feasible or clinically meaningful remains to be established and would require dedicated prospective studies.

Box 3 Center-specific implementation protocol Based on the findings of this study, accurate iodine and iron quantification using PCD-CT requires scanner-specific calibration, even for a scanner from the same vendor. To facilitate the adoption of the proposed correction method, the following protocol is recommended for centers to determine center-specific parameters

**Step 1: Phantom preparation**

Use a spectral CT phantom with inserts containing known concentrations of iodine (in water-equivalent material) and iron (in liver-equivalent material). Scanning with and without a fat ring is recommended to simulate different patient sizes.

**Step 2: Image acquisition**

Scan the phantom using the same protocols intended for clinical use, varying:

- Tube voltage
- Dose
- Phantom size (with and without fat ring)

Ensure consistent reconstruction settings. Save data in a format that retains spectral information.

**Step 3: ROI placement and CT number extraction**

Draw ROIs in each insert on both low- and high-energy images. Record mean low- and high-energy HU values for each concentration and configuration.

**Step 4: Base material CT number calculation**

Plot low- and high-energy HU values versus material concentration and perform linear fits. The y-intercepts represent high- and low-energy base material CT numbers; their difference defines ${\Delta }_{{base}}$.

**Step 5: DER calculation**

Plot high-energy HU values against low-energy HU values for each configuration. The slope of the linear fit is the ${DER}$ for that configuration.

**Step 6: Concentration scaling factor**

Use corrected CM values (adjusted for ${\Delta }_{{base}}$ and ${DER}$) and plot them against known concentrations. The slope of this fit gives the concentration scaling factor $\alpha$.

Equation 2 is used to compute corrected CM values.

**Step 7: Method construction**

For each tube voltage and material, average the values of ${DER}$, ${\Delta }_{{base}}$, and $\alpha$ across all scan configurations. These define the site-specific “mean method”.

**Step 8: Integration into clinical workflow**

- In the material decomposition software, input the site-specific ${DE}{R}_{{avg}}$ to generate CMs.
- Apply the mean method to convert the $C{M}_{{measured}}$ values to concentration (mg/mL).

## Data Availability

The datasets used and/or analyzed during the current study are available from the corresponding author on reasonable request.

## Abbreviations

CM: Concentration map
DER: Dual-energy ratio
HU: Hounsfield units
PCD-CT: Photon-counting detector CT
ROIs: Regions of interest
